# Views held by South African primary health care nurses on caring for people living with mental illness

**DOI:** 10.4102/sajpsychiatry.v30i0.2148

**Published:** 2024-05-03

**Authors:** Nokukhanya Phungula, Lesley Robertson, Sumaya Mall

**Affiliations:** 1 Department of Psychiatry, Faculty of Health Sciences, University of the Witwatersrand, Johannesburg, South Africa; 2 Department of Public Health, Faculty of Health Sciences, University of the Witwatersrand, Johannesburg, South Africa

**Keywords:** PLWMI, task shifting, PHCNs, integrated mental healthcare, nurses’ views on mental healthcare, South Africa

## Abstract

**Background:**

Primary healthcare is the first point of entry into the healthcare system. Scaling up primary mental healthcare is recommended in South African mental health policy. However, there is a paucity of data exploring the views of primary healthcare nurses (PHCNs) with regards to caring for people living with mental illness (PLWMI) in South Africa.

**Aim:**

To explore the views of PHCNs around caring for PLWMI and task shifting.

**Setting:**

A community health centre in Gauteng province, South Africa.

**Methods:**

A qualitative study design using the framework approach was employed. Semi-structured individual interviews were conducted among a convenient sample of PHCNs in a community health centre in Gauteng. Interviews were transcribed and data analysed thematically.

**Results:**

Eight PHCNs were interviewed in June 2022. Five themes emerged: (1) participants highlighted their current practice which excludes mental healthcare; (2) participants described feeling fearful of caring for PLWMI; (3) participants ascribed their lack of confidence in caring for PLWMI largely due to insufficient under- and post-graduate mental healthcare training. (4) task shifting was not welcome due to inadequate mental healthcare training and preexisting challenges in the healthcare system; and (5) recommendations to prioritise mental healthcare training prior to implementing task shifting were made.

**Conclusion:**

Primary healthcare nurses, although empathic towards PLWMI, expressed discomfort with caring for them. This is influenced by multiple factors, some of which may be addressed by improved training and support.

**Contribution:**

This study provides insight into how PHCNs feel about caring for PLWMI and task shifting.

## Introduction

A global commitment towards improved mental health and well-being was made in the United Nations Sustainable Development Goals.^1^ However, in South Africa, limited mental healthcare services contribute to a high treatment gap of close to 92% for mental disorders.^2^ One way of increasing mental health coverage is through ‘task shifting’, whereby services normally delivered by mental health professionals may be delivered by nonspecialist providers such as primary healthcare nurses (PHCNS), who form the ‘backbone’ of primary health care.^3,4^ In addition, even if not providing specific mental health interventions, PHCNs do have to provide general primary healthcare to people living with mental illness (PLWMI).

### Task shifting

In South Africa, task shifting of certain medical practitioner duties to PHCNs has increased access to antiretroviral therapy and to treatment of noncommunicable diseases such as diabetes and hypertension.^5,6^ Task shifting for mental healthcare was also recommended by the South African National Mental Health Policy Framework and Strategic Plan 2013–2020.^7^ Accordingly, mental healthcare is included in the Adult Primary Care (APC) treatment guidelines and the National Department of Health Primary Health Care Standard Treatment Guidelines (NDOH STGs).^6^

Primary health care nurses are professional nurses with an additional qualification in primary care nursing (Academy of Science of South Africa [ASSAF]).^4^ While training as a professional nurse tends to differ across South Africa with respect to exposure to psychiatric mental health training, the new standardised curricula have no exposure to psychiatry.^4^ However, the postgraduate PHCN course is based on the APC guidelines which does include primary mental healthcare (ASSAF).^4^ Primary health care nurses are expected to conduct physical assessments, prescribe medication up to schedule 4 and refer for further treatment. The APC guidelines expect competence in assessments of aggressive patients and those suffering from substance abuse, depression, anxiety, psychosis and dementia, familiarity with the mental healthcare act and ability to refer appropriately.^5^ However, a consensus study by the ASSAF found mental health training modules for PHCNs to be inadequate in terms of the expected core competencies based on the APC guidelines.^4,5^

Globally, nursing staff have been found to hold pessimistic views on the prognosis and outcomes of mental illness and to display discriminatory behaviour towards PLWMI.^8,9,10^ Negative attitudes towards PLWMI may contribute to suboptimal therapeutic relationships and may exacerbate delays in help-seeking and poor treatment adherence, resulting in poor-quality mental and physical care.^9^ Nevertheless, skilled base training, workshop-based interventions and intensive social contact interventions may improve empathy, reduce anxiety and increase the understanding of PLWMI among nurses. Behaviour change taught in skill-based interventions can increase comfort and confidence while also expending on understanding of mental illness as being manageable.^9^

### Problem statement

In South Africa, the majority of PLWMI do not get the care that they require. Task shifting, using PHCNs to provide some of the care traditionally provided by medical practitioners or specialists, may help to close the treatment gap.^2^ In addition, empathetic and positive attitudes towards PLWMI in general are important in maintaining adherence and improving help-seeking behaviours.^7^ However, concerns have been raised regarding training inadequacies for PHCNs.^4^ It is therefore important to evaluate the views of PHCNs towards caring for PLWMI and task shifting.

### Aim and objectives

This study aimed to investigate the views held by PHCNs regarding caring for PLWMI and task shifting. The objectives were to explore PHCNs’ views on providing mental healthcare and their recommendations as to what strategies and interventions may assist them in caring for PLWMI.

## Research methods and design

### Study design

A qualitative study design using the framework approach was employed.^11^ The principal investigator (NP) was a registrar in psychiatry. Coauthors S.M. and L.R. have extensive experience in qualitative research and in public mental health, respectively, and supervised the study.

### Study setting

The research was conducted at a Community Health Centre (CHC) which serves a township population in Gauteng province. This CHC was purposefully selected as, when obtaining permission from the study site, the researcher was informed by the supervisor that the staff at this CHC are usually willing to participate in research. Services offered at the CHC include a 24 h emergency casualty, 24 h maternity unit and an outpatient clinic for communicable and noncommunicable chronic diseases. Allied health services include social worker, occupational therapy, optometry and limited radiographic, dental and dietetic services. There is also a coronavirus disease 2019 (COVID-19) vaccination programme available that offers both COVID-19 testing and vaccination.

Regarding mental healthcare, the CHC has a community psychiatric clinic which is supervised by a psychiatrist and run by two PHCNs with psychiatric nursing experience. People living with mental illness are attended to by doctors undergoing psychiatric training, clinical psychologists and occupational therapists. Each discipline provides a service on 2 days of the week (1 day for children and adolescents, and 1 day for adults). The community psychiatric clinic takes referrals from PHC and inpatient psychiatric units. In turn, PLWMI whose conditions are mild or stable are referred from the psychiatric clinic to PHC for ongoing care. Thus, the PHCHs are expected to identify, provide immediate containment, refer appropriately and provide and continue chronic care for stable patients. The service is described in more detail by Robertson and Szabo.^12^ Including the mental health service, the CHC has 36 nurses (of whom 17 are PHCNs and the rest are registered nurses and professional nurses), who serve approximately 6000 people per month.

### Study sample

The study population comprised of participants (information masked for blind review) working at the CHC in June 2022. This population was suitable for the study as nurses are often the first point of care for PLWMI. As task shifting aims to increase access to mental healthcare, PHCNs would be able to diagnose mental illness and continue treatment as their qualification is more specialised. People living with mental illness would then be able to receive more coverage in terms of care outpatient and also possibly decrease institutionalisation. To be included, the PHCNs had to have a qualification in primary health care nursing, work in any service provided by the CHC, except for the community psychiatric clinic, and consent to the study. The two PHCNs running the specialist mental health service were excluded from the study as they already provide mental health services at specialist level. Prior to the interviews, the investigator and research topic were introduced to the PHCNs by the investigator. Both purposive and convenience samples of PHCNs on day duty during the study period were used. The PHCNs initially on night duty were later interviewed when they were on day duty. Recruitment of participants continued until data saturation was reached. Data saturation was determined when it became evident that similar views were being repeated by subsequent participants and that no new information relevant to the research question was emerging.

### Sample size

Ten nurses were approached and agreed and consented to participate. However, only eight were interviewed, as two nurses were unavailable at the time of the interviews. As the eight participants all expressed similar views, further participants were not recruited.

### Data collection

All data were collected by one investigator using a voice recorder. The investigator had no relationship with the participants. Semi-structured, individual in-depth interviews were conducted over a 3-week period in June 2022. On average, each interview lasted 40 min – 60 min and was conducted in English and isiZulu according to the participants’ preference. To ensure that the views of the participants were captured accurately, notes and comments were reviewed with the participant near the end of the interview.

### Interviews

The interviews conducted consisted of a dialogue between the participants and researcher in a confidential space. Open-ended questions were asked, with time allowed for answers, enabling exploration of the participants’ experiences and ideas. Prompting, using a topic guide with follow-up questions, encouraged more in-depth responses and assisted in keeping the discussion on the topic. The topic guide included getting to know the participant in terms of their training in, understanding of and contact with PLWMI. Moreover, their thoughts on task shifting were unpacked. In addition, views on caring for PLWMI were explored by enquiring about challenges and positive experiences thereof. Furthermore, recommendations on how to assist PHCNs to care for PLWMI were discussed. However, direct questions were avoided in the aim of not limiting the participants’ answers. This allowed the researcher to collect open-ended data and to explore participant thoughts, feelings and beliefs about the topic.

Interviews took place at the CHC in a consultation room which allowed for privacy to be maintained. The intention was that interviewing them in a familiar but private setting would make them feel more comfortable to express their views. The interviews were done at times that did not affect clinical duties or impact service delivery.

### Data analysis

The data were analysed systematically in order to identify and interpret themes and subthemes as described by Hackett and Strickland.^13^ Each interview was transcribed verbatim (with translation into English when the recording was in isiZulu) for later thematic analysis from the recordings. The principal investigator familiarised herself with the data by reading and rereading the transcripts, while being attentive to emerging patterns. Codes were generated to document how these patterns occurred. These codes were subsequently combined to form different themes, which were examined further to ascertain how they support the data. Themes which made meaningful additions to understanding what the data revealed were highlighted. Finally, to lessen the possibility of misinterpretation, the data were revisited to guarantee that the participant accounts were presented correctly. To minimise bias, three transcripts were read by all three authors independently, who met thereafter and compared their findings.^13^

### Study rigour

The data were read and reread while being attentive to the themes that emerged. Codes were generated to document where and how these themes occurred. In order to lessen the possibility of misinterpretation, we revisited the original data to guarantee that the participant accounts were presented correctly.

To ensure objectivity and minimise bias, a random selection of four transcripts was read and coded by both supervisors and the researcher independently. These findings were compared, and consensus was reached regarding the codes and themes identified.

### Ethical considerations

Data were anonymised, and confidentiality was maintained by assigning a study number to each participant. This number was used to label each interview in the recording and the transcript. Permission to conduct the research was provided by the district’s research committee. Ethical approval was obtained from the University of the Witwatersrand Human Research Ethics Committee (reference number M220375).

## Results

The eight participants (two males and six females) had a mean age of 51 years (ranging from age 39 to 63 years) with an average of 14 years (ranging from 5 to 32 years) of service in primary healthcare. All participants were professional nurses with a PHCN qualification and had been trained on the APC guideline. Two participants had also received additional mental healthcare training in the form of workshops and in-service training. Six participants, including one of the two who had some additional mental healthcare training, had personal contact with PLWMI in the form of a friend, relative or neighbour.

### Emergent themes

Five themes emerged from the data, namely: (1) participants’ description of their current practice regarding mental healthcare, (2) their understanding of and caring for PLWMI, (3) their views regarding their training in mental healthcare, (4) participants expressed their views on task shifting and (5) their recommendations regarding its implementation.

### Participants’ description of their current practice regarding mental healthcare

Participants described that they primarily cared for people with chronic conditions, including HIV and AIDS, tuberculosis (TB), diabetes, hypertension, etc. They did not routinely care for PLWMI. Even when a patient presented with comorbid mental illness, priority was given to the physical condition, as Participant 2 explained: ‘Even if a TB patient is also a mental healthcare user, I just focus on the TB part’:

Those presenting with symptoms of mental illness were immediately referred to the mental healthcare clinic to be seen by psychiatry doctors. This was reflected by the following quotes:

‘[… *W*]e have a separate mental side. When they come, they go straight to mental health clinic.’ (Participant 3, age 55, female)

‘[*T*]he mental health care users [*MHCUs*] go straight to the mental health clinic; we don’t see them.’ (Participant 8, age 63, female)

However, afterhours, all PHCNs have to care for PLWMI presenting to the emergency room. This resulted in feelings of fear as Participant 7 expressed that ‘containing these patients needed the assistance of security guards’.

In addition, PHCNs expressed not knowing their roles in caring for PLWMI as evidenced by the following:

‘[*I*]n a case where there is no doctor you have to ask around on how to help this patient because there’s no document or protocol or something.’ (Participant 2, age 52, male)

‘[*T*]here’s no tools you just ask around.’ (Participant 3, age 55, female)

‘I really don’t know but also I think it’s because there are already nurses trained in that who are working in mental health.’ (Participant 6, age 46, female)

### Participants’ views on caring for people living with mental illness

Overall, the participants viewed PLWMI as being ‘unpredictable’ with a potential for aggression and violence. In addition to feeling unsafe around PLWMI, the participants were reluctant to care for PLWMI, with Participant 6 explaining: ‘These patients are too different for me. There are those that I’m afraid of …’.

The participants displayed conflictual feelings between their fears of caring for PLWMI and a sense of duty to care for them. Participant 2 reported: ‘It’s a very mixed feeling. I feel scared and want to run but at the same time I must also attend to these patients’ *and* another participant expressed feeling ‘lucky’ to have a specialised mental healthcare service available at the facility.

Probing regarding caring for people with nonpsychotic illness such as depression revealed a sense of impatience and intolerance. Some participants minimised depression calling it ‘just stress’. Participants 3 and 8 were dismissive, expressing that ‘even us as nurses we are depressed’ and Participant 8 described having reprimanded a suicidal patient by saying ‘what’s your problem?’ ‘I’m taking medication but do you ever hear me saying I’m going to kill myself?’

Notwithstanding the fearfulness, a desire to escape the duty to care and impatience with nonpsychotic illness, compassion for PLWMI was evident. Several participants emphasised that PLWMI are ‘human first before their condition’:

‘[*J*]ust because someone has a mental illness it doesn’t mean they are not human.’ (Participant 1, age 62, female)

‘Mental illness is not something they chose.’ (Participant 3, age 62, female)

‘They are human first before their condition.’ (Participant 5, age 39, male)

‘[*T*]hey need patience and time. They want to be listened to. They want to be treated as human.’ (Participant 8, age 63, female)

### Participants’ views on their previous training in mental health

Most participants could not recall having had any undergraduate mental healthcare training, even though it was part of the curriculum. Others expressed it was insufficient, with Participant 1 confessing that ‘it was meant to be of six months’ duration but was shortened’; Participant 7 added ‘they would not let us interact with the patients’.

Lack of training continued post-qualification while there was ‘constant training’ (Participant 2, age 52, male) in TB and HIV care. Poor training appeared to have a negative impact on how PHCNs view and care for PLWMI. Three participants noted a difference between nurses who had some post-qualification mental healthcare training and experience and those who had not. The older participants felt that the younger nurses were ‘not equipped’ to care for PLWMI as they ‘just don’t have the knowledge’ (Participant 1, age 62, female) and ‘they don’t have the experience’ (participant 8, age 63, female). Discriminatory behaviour towards PLWMI was described by one participant:

‘The nurses with no psychiatric training are very afraid of the patients. The younger nurses would not attend to them, or they would put their files aside. The nurses are very irritable towards these patients.’ (Participant 1, age 62, female)

### Participants’ views on task shifting

Six of eight participants expressed being against task shifting. They complained of already being overwhelmed with service load, being understaffed and lacking the capacity to attend to the special needs of PLWMI, who they viewed as requiring exclusive attention. Furthermore, the participants felt that PHC infrastructure and clinic systems were inadequate to cater for PLWMI. While one of the older nurses considered that ‘task shifting may reduce how much patients get dismissed by nurses when they come to PHC’ (Participant 1, age 62, female), others felt that task shifting would have a negative impact on PLWMI. Participants’ views included:

‘[*PLWMI*] are going to come here and see an unfriendly tired face … some patients stop taking their treatment because some of us shout at them … we feel overburdened and overwhelmed.’ (Participant 5, age 39, male)

‘Logistically it’s not possible. The clinic is very, very busy. There will be extra workload.’ (Participant 6, age 46, female)

A sense of feeling abused by policy makers and government, should task shifting be implemented, was also evident. Participant 5 was infuriated by the health system and its demands on nurses expressing the following:

‘[… *N*]urses are treated as scape goats. Whenever the health care system gets stuck, the next line of abuse is a nurse. I’m feeling frustrated about this because in the end we will be forced to comply.’ (Participant 5, age 39, male)

Participant 7 described feeling helpless as their management did not get involved if they attempt to communicate their grievances. Instead, if they complain, management perceives them to be ‘corrupting the whole nursing group’ and is simply given the response of ‘the system is already corrupt’. This left the nurses feeling unheard by their managers and therefore forced to comply with any changes made including task sharing.

On the background of ‘already existing problems’ (Participant 5) in the healthcare system, participants felt the implementation of task shifting would be premature. They listed the existing problems to be poor working conditions, short staffing, lack of medical equipment and consultation rooms to name a few:

‘[*T*]he infrastructure is not going to make it work. Logistically it’s not possible.’ (Participant 6, age 46, female)

‘[*T*]here’s a shortage of nurses and our setting.’ (Participant 3, age 55, female)

‘[*A*]t times you find doctors having to share a room and we also don’t have equipment.’ (Participant 5, age 39, male)

‘We just don’t have space. One of the rooms we had to open as an emergency room because we operate as a 24 hour clinic. So it is a challenge.’ (Participant 6, age 46, female)

### Participants’ recommendations on assisting primary health care nurses to care for people living with mental illness

Prioritising training in mental healthcare was recommended by all participants. This could be done in the form of ‘workshops, in-service training and site visits at psychiatric institutions.’ They felt would improve their skills, attitudes and perceptions of PLWMI which could result in better patient care:

‘I think if they could attend short courses so that they can also be taught that patients are the same as you and I. training should be done before anything is implemented.’ (Participant 1, age 62, female)

‘[*T*]he nursing trade had trained nurses to be empathetic and they now just need stimulation.’ (Participant 2, age 52, male)

‘[*R*]efresher courses will help.’ (Participant 3, age 55, female)

## Discussion

This study revealed several important findings. Firstly, the conversations were dominated by mixed feelings of fear and compassion while also feeling a sense of having a calling to care for people living with severe mental illness or severe behavioural disturbances. Secondly, depression was viewed as something that could be dealt with by the patient. Task shifting was viewed with trepidation with participants’ feeling unequipped and needing more training in mental healthcare prior to implementing this strategy. The study further found that the older nurses had more positive views of caring for PLWMI. This was shaped by having had years of experience working with PLWMI. They thus were more welcoming of task shifting.

### Current practice regarding mental healthcare in PHC setting

The study also found that PHCNs were unaware of their roles in mental healthcare as stated in the APC and NDOH STG’s guidelines. As a consequence, mental illnesses are neglected while other chronic illnesses are prioritised. MHCUs are directed to the mental healthcare clinic, whereas the guidelines state that nurses need to be familiar with and competent in assessment of aggressive patients and those suffering from substance abuse, depression, anxiety, psychosis and dementia (APC).^5^

Of concern, the NDOH referral policy does not include community mental health services, such as the mental health clinic at this CHC.^14^ The referral policy also does not include psychiatric care at district hospital level. Therefore, if the mental health clinic was not available, it is possible that all of the MHCUs attended to at this CHC would have to travel to regional or tertiary hospitals for appropriate care. However, in evaluating integrated mental healthcare in the Kenneth Kaunda District of North West Province, Petersen et al.^15^ found that PHCNS only identified 6% of people with depression prior to training. While the percentage increased to 16% after training, referral to a medical practitioners upskilled for the study was necessary to care.^16^

### Participants’ views on caring for people living with mental illness

Feelings of fear towards caring for PLWMI were highlighted in this study. This was in keeping with most of the participants feeling scared to care for PLWMI. They expressed having a desire to help them, however, feeling unequipped due to lack of training in undergrad and post-qualification. Furthermore, they understood them as being a special group with their own staff. In addition to this, most participants were not interested in mental health. Those that were, were struggling to get further training in mental health.

### Participants’ views on their training needs

The overwhelming gaps in training, which also exist post-qualification, have led to PHCNs expressing feelings of being unequipped to care for PLWMI. The older nurses expressed concern as they have noted these gaps in training worsening over the years. This has resulted in the younger nurses having a lack of understanding of mental healthcare. This is supported by findings by the ASSAF report which suggest that psychiatric training has been excluded in the nursing curriculum.^4^ Moreover, the new nursing curriculum, although making a promise to give attention to psychosocial issues and to PLWMI, is unclear on the details of this. The South African Nursing Council outlines the bachelor’s nursing degree as being inclusive of integrating assessments of those living with mental illness.^4^ However, again, lacking detail. The ASSAF report further highlights this gap in training as being the source of nurses feeling uncomfortable to care for PLWMI.^4^ Expecting them to then have positive views towards caring for PLWMI would be unfair without this foundation and further training post-qualification.

This finding is comparable to that of a study by Lien et al.^17^ This study, by Kluit, described lack of contact with those living with mental illness and inadequate education about mental illness as being important reasons for negative attitudes towards PLWMI. Mental healthcare training is seen as an option and further training is flawed. This further impacts negatively on patient’s adherence causing them to relapse, giving them bad attitudes, being impatient with them and dismissive. Likewise, Knaak et al. suggested that these negative attitudes towards mental illness among healthcare practitioners create barriers to care, including delays in help-seeking behaviour, nonadherence to treatment, suboptimal therapeutic relationships and poor-quality mental and physical care.^9^

### View on integrated primary health through task shifting

While the nurses in this study acknowledged that PLWMI are human first before their illness, this did not translate into them wanting to care for them through task shifting. It seemed as though the negative experiences, mostly ones that created feelings of fear, of PLWMI outweighed their sense of duty to care for PLWMI. It was evident that their lack of competence may be the driving force behind PLWMI.^18^

In addition, feelings of having lack of support and training increased PHCNs’ reluctance to care for PLWMI. This finding is similar to that of Janlov et al.^19^ who identified that nurses described feeling ‘insecure’ regarding how to deal with PLWMI due to lack of knowledge.

Task shifting alarmed PHCNs. This is especially because, although task shifting is a government policy, there is no national guideline in terms of its implementation. Furthermore, the WHO guidelines on task shifting do not put emphasis on or give priority to mental healthcare. Rather, HIV is the focus. The participants expressed grievances of multiple possible challenges with this concept which aggravated the preexisting issues they have with government. This further fuelled the decision to dismiss the idea of task shifting based on staff shortage and therefore feeling overwhelmed. Moreover, the primary healthcare setting is already chaotic which may be an aggravating environment for PLWMI. In addition, nurses play multiple roles. Lastly, the lack of support and training from managers was also mentioned as a reason for task shifting not being embraced.

Task shifting is posed as a very positive solution to increasing accesses to mental healthcare.^7^ However, Hanlon et al.^20^ found that it needs ongoing training and supervision. In addition, in this study by Hanlon et al., which was done in Ethiopia, surprisingly task shifting was not cheaper and did not increase adherence although being community based.^20^

The participants’ frustration towards task shifting was fuelled by feeling unprioritsed and their concerns not advocated for by managers and policy makers. This is validated by Thornicroft et al. who found that LMICs such as South Africa are not yet prepared for task shifting to be implemented.^21^

### Views on challenges with task shifting

Primary health care nurses own mental health may be compromised. This may manifest negatively as burnout and compassion fatigue among healthcare providers which may exacerbate stigmatisation of PLWMI.^9^ This is similar to the study by Koutra et al.^22^ who found that emotional exhaustion, depersonalisation, compassion, fatigue and burnout are linked to negative attitudes towards PLWMI.

### Views on decisions made by policy makers

Policy makers were seen to be inconsiderate, corrupt and displaying leadership skills which make caring for PLWMI a near impossible task to achieve well. As a result of this, the idea of task shifting was largely dismissed.

### Recommendations on assisting primary health care nurses to care for people living with mental illness

While all nurses recommended to have training workshops or short courses in mental health, some were still not in favour of task shifting. They felt the training would help them care for PLWMI better and consider task shifting in the future. The recommendation for training is consistent with the recommendations made by Basson, Hester and Adejuma^23^ and (Nyblade et al.^24^), who emphasised the need for decreasing negative attitudes and stigma towards PLWMI. They suggested that this could be achieved by educating and equipping healthcare providers with regards to mental conditions and stigma. Furthermore, contact with stigamtised individuals could may break stereotypes and encourage empathy.

### Limitations

Some limitations are noted in the study. Due to the participant being a registrar in psychiatry, the participants may have had the perception that the investigator is in favour of task shifting and integration of patients into primary healthcare. The interviews were only conducted at one CHC; therefore, the finding may not be transferable to other PHCNs in South Africa. Due to the chaotic nature of the clinic, some interviews were interrupted or did not occur on time. The chaotic nature was seen through not having enough consultation rooms to conduct the interviews. Moreover, the nurses would sometimes be requested to go assist in another section of the clinic due to ongoing staff shortages.

### Strengths

The semi-structured interviews allowed for challenges regarding caring for PLWMI and task shifting to be explored in detail and depth. Important insights into the participants’ thoughts and feelings were learned. This can inform managers and policy makers on more effective interventions to address the challenges mentioned.

### Recommendations

Improving and prioritising mental healthcare training was highly recommended by the PHCNs in this study. This can be in the form of incorporating mental healthcare training in the nursing curriculum, workshops, short courses and by also increasing contact with PLWMI in the form of site visits to psychiatric institutions. Lastly, more research needs to be conducted with regards to views of nonspecialists on caring for PLWMI in line with mental healthcare being a global priority. Policy makers and managers should be made aware of the challenges experienced by PHCN when it comes to caring for PLWMI and task shifting. Moreover, the government should attempt to consult and include PHCNs when making decisions or implementing changes in the healthcare system.

## Conclusion

The views of selected PHCNs at a Gauteng Province CHC suggest that nurses acknowledge the duty and may have the desire to provide care for PLWMI. However, they may not welcome the concept of task shifting. Their reluctance was influenced by previous negative experiences in caring for PLWMI and a lack of training in mental healthcare. Moreover, nurses being excluded from decisions made by policy makers may have fuelled this resistance.
